# Optimized Sulfonated Poly(Ether Ether Ketone) Membranes for In-House Produced Small-Sized Vanadium Redox Flow Battery Set-Up

**DOI:** 10.3390/membranes14080176

**Published:** 2024-08-14

**Authors:** Antonino Rizzuti, Elena Dilonardo, Gennaro Cozzolino, Fabio Matera, Alessandra Carbone, Biagia Musio, Piero Mastrorilli

**Affiliations:** 1 Department of Civil, Environmental, Land, Building and Chemical Engineering (DICATECh), Politecnico di Bari, Via E. Orabona 4, 70125 Bari, BA, Italy; 2 Institute of Nanotechnology, CNR-NANOTEC, Via G. Amendola, 122, 70125 Bari, BA, Italy; 3 InResLab, Via Baione, 70043 Monopoli, BA, Italy; 4 Institute of Microelectronics and Microsystems, CNR-IMM, Catania HQ, VIII Strada n. 5, 95100 Catania, CT, Italy; 5 Institute for Advanced Energy Technologies, CNR-ITAE, Salita S. Lucia Sopra Contesse 5, 98126 Messina, ME, Italy

**Keywords:** ion-selective membranes, sulfonated poly(ether ether ketone) membrane, degree of sulfonation, SPEEK/amino–silica hybrid membrane, vanadium redox flow battery, chloride-based electrolyte, coulombic efficiency, charge–discharge analysis

## Abstract

The ionic exchange membranes represent a core component of redox flow batteries. Their features strongly affect the performance, durability, cost, and efficiency of these energy systems. Herein, the operating conditions of a lab-scale single-cell vanadium flow battery (VRFB) were optimized in terms of membrane physicochemical features and electrolyte composition, as a way to translate such conditions into a large-scale five-cell VRFB stack system. The effects of the sulfonation degree (SD) and the presence of a filler on the performances of sulfonated poly(ether ether ketone) (SPEEK) ion-selective membranes were investigated, using the commercial perfluorosulfonic-acid Nafion 115 membrane as a reference. Furthermore, the effect of a chloride-based electrolyte was evaluated by comparing it to the commonly used standard sulfuric acid electrolyte. Among the investigated membranes, the readily available SPEEK50-0 (SD = 50%; filler = 0%) resulted in it being permeable and selective to vanadium. Improved coulombic efficiency (93.4%) compared to that of Nafion 115 (88.9%) was achieved when SPEEK50-0, in combination with an optimized chloride-based electrolyte, was employed in a single-cell VRFB at a current density of 20 mA·cm^−2^. The optimized conditions were successfully applied for the construction of a five-cell VRFB stack system, exhibiting a satisfactory coulombic efficiency of 94.5%.

## 1. Introduction

Nowadays, the global warming emergency and the increase in energy demands lead the global interest in renewable energy sources such as solar and wind power. However, their intermittent supply requires the development of efficient and low-cost energy storage systems (ESSs) [1,2,3]. In this context, redox flow batteries (RFBs) are suitable candidates for energy storage thanks to their capability to decouple capacity and power, their high efficiency, good reliability, high design flexibility, rapid response, and long cycle life [4]. Among the great variety of RFBs, vanadium redox flow batteries (VRFBs) are the most promising commercial large-scale hybrid power systems. Indeed, VRFBs show a fast response to energy demand change, high energy efficiency, low environmental impact, long life cycle, flexible design, and low installation cost [5,6,7,8].

The electrodes and the ion exchange membranes are essential components in VRFBs because their morphological, structural, chemical, and physical properties affect the overall performance, durability, and efficiency [7]. Furthermore, the electrolyte is a key element for VRFB performance, influencing energy efficiency and cycle duration, as well as stability and conductivity [9]. Although VRFBs have attractive features, their widespread commercial diffusion and usage are still greatly hindered by the high capital cost of the ion exchange membranes, the fluctuation in the price of electrolytes, and the low energy and power density [7,10]. Therefore, great efforts of the current research are devoted to the development of materials to be used as alternative electrodes, electrolytes, and membranes to reduce costs and improve the comprehensive performance of VRFBs [5,11]. An ideal electrode for VRFB application should have high electrical conductivity, high specific surface area, and high chemical stability. Such features are well met by carbon-based electrodes, e.g., graphite or carbon felt [12,13,14]. However, large-scale application of graphite felt (GF) is limited by its poor hydrophilicity and electrochemical activity; hence, tremendous efforts have been devoted to improving the electrochemical properties of GF. The modification of GF is mainly achieved by increasing the amount of active functional groups, enhancing the effective area, and introducing surface catalysts [15].

One of the VRFB advantages is the possibility to overcome potential issues derived from cross-contamination through the membrane, which is a typical issue in flow batteries [16,17,18]. Indeed, in VRFBs, vanadium serves as both the cathodic and anodic electrochemical active species, utilizing VO_2_^+^/VO^2+^ and V^3+^/V^2+^ redox couples as positive and negative electrolytes, respectively. In such circumstances, the efficiency loss caused by vanadium crossover can be straightforwardly mitigated by appropriate rebalancing of the electrolytes. Furthermore, in VRFBs, vanadium electrolytes which function as both the electrolyte and active material are highly important in terms of the overall cost and performance [19]. Specifically, the electrochemical activity, concentration, and stability of vanadium ions affect the energy density and reliability of VRFBs [20]. Although VRFB electrolytes have been improved during the last few decades [21], a continuous effort is necessary to improve vanadium solubility, stability, and electrochemical performance for the design of energy-dense, reliable, and cost-effective VRFB technology [22]. In our previous study [9], a chloride-based electrolyte was efficiently prepared by V_2_O_5_ chemical reduction in the presence of oxalic acid, and successfully employed in a single-cell VRFB. This adopted preparation method of the electrolyte, which starts from the readily available V_2_O_5_, has been reported to be easily implemented on an industrial scale [23,24,25].

The ion exchange membrane (IEM) is one of the key components of a common VRFB. It allows the separation between the positive and the negative electrolytes, keeping the protons’ transfer active to close the electrical circuit. IEMs have a deep impact on the performances (e.g., efficiency, cycling stability, capacity fading, etc.) and the system cost (up to 30–50%) [26]. An ideal IEM is expected to have high chemical stability, mechanical strength, satisfactory proton conductivity, and a low crossover of vanadium ions through the membrane, which separates the anolyte and catholyte to avoid electrolyte imbalance [27]. Currently, perfluorinated Nafion membranes are widely used in VFRBs since they meet the requirements mentioned before [28]. Nevertheless, their high-cost and high vanadium permeability may reduce the battery performance, hindering large-scale application and commercialization [16]. Therefore, great efforts have been made to replace Nafion with alternative non-perfluorinated-based IEMs to overcome such limitations [29,30,31,32]. Among all possible candidates for IEMs [28,33], functionalized poly(ether ether ketone) (PEEK) membranes containing sulfonic groups (SPEEK) [34,35] have been reported as promising materials to replace Nafion. Such membranes are characterized by high stability, low-cost [16,36], and high proton-to-vanadium ion selectivity [34,37,38]. Additionally, their preparation is straightforward with a tunable sulfonation degree (SD) [27,39], which is generally modulated based on the final application [39]. Generally, a high SD causes high water uptake, and, consequently, an excessive swelling and even dissolution of the membrane [26]. Therefore, a relevant decrease in the chemical and mechanical stability of the membrane is observed, with a significant penetration of vanadium ions [40]. At the same time, SPEEK membranes with low SD have a too-unsuitable proton conductivity which hinders proton transport through VRFBs [41]. Therefore, much effort has been devoted to increasing the proton conductivity of SPEEK materials, improving the chemical and mechanical stability of the membranes [33,34]. In this context, covalent modification, cross-linking, hybridization, and composite formation with functionalized inorganic nanomaterials have been explored [27,34,39,42,43]. Furthermore, the addition of inorganic fillers (e.g., SiO_2_, TiO_2_, WO_3_) has been exploited to increase the operating temperatures compared to the pure polymer [44,45,46]. Another reported approach to improving the temperature stability of SPEEK membranes by limiting the loss of proton conductivity is the development of blended membranes containing both sulfonic and nitrogenous groups, utilizing the different acid strengths of the two different functionalities [30]. Hybrid SPEEK membranes filled with 3-aminopropyl functionalized silica gel have been tested, showing improved mechanical resistance and proton conduction. Indeed, the interactions between the sulfonic groups of the polymers and the aminic groups in the silica gel affected both the swelling phenomena at critical high temperatures and the reduction of the proton conductivity [44,47]. Silica nanoparticles functionalized with the 3-aminopropyl group have also been introduced as filler in Nafion membranes to improve the long-term performance of a VRFB [48].

Based on these encouraging reported results, we undertook research to explore the feasibility of the SPEEK membranes at various SDs, and filled them with SiO-NH_2_ filler, moving towards the development of an in-house-assembled small-sized VRFB system. We investigated the chemical–physical and electrochemical properties of the prepared SPEEK membranes in a lab-scale single-cell VRFB in the first instance, comparing the obtained performances to those of the commercial Nafion 115 membrane. Furthermore, the effect of a chloride-based electrolyte was investigated over the standard sulfuric acid one. Higher energy density could be reached in the presence of a chloride-based electrolyte, thanks to the higher solubility of vanadium in it, compared to standard sulfuric acid. Moreover, under the operation conditions, the use of a chloride-based electrolyte allowed us to obtain a minor resistance of the system, and a better voltage efficiency. The optimal membrane was selected and subsequently employed in combination with a chloride-based electrolyte to produce a VRFB system made up of a five-cell stack. The performance of the resulting VRFB system was assessed through consecutive charge–discharge cycles and electrochemical impedance spectroscopy (EIS) measurements.

## 2. Experimental Details

### 2.1. Materials

PEEK was purchased from Victrex in the form of a fine powder (450PF); sulfuric acid (H_2_SO_4_ 96%_v_) was purchased from the Carlo Erba Reagents Srl (DASIT Group, Emmendingen, Germany); and dimethylacetamide solvent (DMAc 99%_v_) and 3-aminopropyl functionalized silica gel (~1 mmol g^−1^ NH_2_ loading) were purchased from the Sigma-Aldrich company (Merck KGaA, Darmstadt, Germany).

V_2_O_5_ (provided by Duferco Energia SpA (Genoa, Italy), 99.95%), VOSO_4_·2H_2_O (Sigma-Aldrich, 97%), HCl (Sigma-Aldrich, 37%_w_, d = 1.20 g/mL), H_2_SO_4_ (Sigma-Aldrich, 98%_w_, d = 1.84 g/mL), and C_2_H_2_O_4_·2H_2_O (Sigma-Aldrich, 99.6%) were used as received. A standard sulfuric acid VRFB electrolyte (STD_Electrolyte), nominally composed by [V^3+^] = 0.75 M, [VO^2+^] = 0.75 M, and [SO_4_^2−^] = 3.00 M with a [V^3+^]/[VO^2+^] ratio equal to 1, was purchased from C-Tech Innovation Ltd. (Chester, UK).

Perfluorinated membrane, Nafion 115 (thickness 0.005 in.), was purchased from DuPont™ (DuPont Fluoroproducts Fayetteville, Greensboro, NC, USA). Carbon felt electrode, GFD 4.6EA, was obtained from SGL Carbon (Meitingen, Germany).

### 2.2. Preparation of SPEEK Powders

SPEEK polymers (Supplementary Information, Figure S1a) at 50% and 64% SD (SPEEK50 and SPEEK64) were prepared by sulfonating PEEK (Supplementary Information, Figure S1b) at temperatures of 30 °C and 35 °C, respectively, for 24 h, following the procedure described in Ref. [49].

Elemental analysis for the calculation of SD and structural characterization of prepared membranes were performed as reported in Refs. [50,51].

### 2.3. Preparation of Membranes

SPEEK polymers were used to obtain the correspondent membranes, SPEEK50-0 and SPEEK64-0, by casting on a glass sheet (doctor-blade technique) a dispersion of 6–10%_w_ SPEEK polymers in DMAc [49]. Composite membranes (Supplementary Information, Figure S1c) were obtained by mixing different weight percentages of 3-aminopropyl functionalized silica gel with the polymer dispersion before the casting step. Then, 10%_w_ and 20%_w_ percentages of 3-aminopropyl functionalized silica gel were used in both SPEEK membranes, as follows: SPEEK50-10, SPEEK50-20, SPEEK64-10, and SPEEK64-20. The composite SPEEK membranes were dried at 80 °C for 3 h and at room temperature for about 16 h to eliminate the residual solvent, washed in water, and treated with acid to purify the obtained films.

Scaled-up membranes of a 19 cm × 29 cm dimension were prepared for stack realization.

The perfluorinated Nafion 115 membrane (Supplementary Information, Figure S1d) was used as a reference.

### 2.4. Chemical–Physical Characterization of the Membranes

The membrane thickness was measured by using a thickness gauge Mod. ID-C112PB (Mitutoyo Corp., Kawasaki, Japan).

The ion exchange capacity (IEC) of the membranes was calculated by determining the equivalent point of an acid–base titration, by using an automatic titrator (Mod. 751GPD Titrino, Metrohm AG, Herisau, Switzerland) and NaOH 0.01 M (Carlo Erba, Normex^®^) as a titrant [52].

The water uptake (W_up_) and the vanadium uptake (V_up_) percentages were calculated as reported in the following Equations (1) and (2):

(1) $$\%W_{up}=\frac{m_{wet}-m_{dry}}{m_{dry}}\times 100$$

(2) $$\%V_{up}=\frac{m_{wet}-m_{dry}}{m_{dry}}\times 100$$

where m_dry_ and m_wet_ are the dried and wet weight, respectively, of membranes. Specifically, the dry mass was obtained after drying the membrane in an oven under vacuum for 2 h at 80 °C; the wet mass was obtained after immersing the dried membrane in water for the W_up_, or in an aqueous solution of VOSO_4_ 1.0 M and H_2_SO_4_ 2.0 M for V_up_, at room temperature for 24 h [51].

The vanadium ion permeability ($P_{{VO}^{2+}}$) of the membranes was measured using a two-chamber diffusion cell. One chamber was filled with 1.0 M VOSO_4_ solution in 2.0 M H_2_SO_4_ (reservoir A); the other chamber was filled with 1.0 M MgSO_4_ solution in 2.0 M H_2_SO_4_ (reservoir B) to nullify the effects of osmotic pressure [8]. In the reservoir B solution, the concentration of VO^2+^ was measured by a UV-Vis spectrometer at 765 nm wavelength. The vanadium ion permeability ($P_{{VO}^{2+}}$)) was calculated using the Equation (3), as follows:

(3) $$P_{{VO}^{2+}}=\frac{V_{B}L}{A[C_{A}^{0}-C_{B}\left(t\right)]}\cdot \frac{dC_{B}(t)}{dt}$$

where V_B_ is the volume of reservoir B; L and A are the thickness and area (3.14 cm^2^) of the membrane; C_A_^0^ and C_B_(t) are the initial VO^2+^ concentration in reservoir A and the concentration of VO^2+^ in reservoir B at time t, respectively [53].

The ionic conductivity (σ) of the membranes was measured in the longitudinal direction using a four-electrodes method and DC, by using a commercial conductivity cell (Bekktech LLC, Loveland, CO, USA) and a potentiostat-galvanostat (mod. 551 AMEL, S.r.l., Milan, Italy), as reported in Ref. [51]. A 2 cm × 3 cm membrane sample was assembled into the cell in contact with two platinum electrodes placed at a fixed position and fed with humidified H_2_. σ was determined at 30 °C and 100% R.H., before and after the vanadium uptake measurements, using the following equation:

(4) $$\sigma =\frac{0.425}{w\;t\;R}$$

where 0.425 cm is the fixed distance between the Pt electrodes, *w* is the width of the membrane, and *t* is the thickness, both expressed in cm, while *R* is the membrane resistance [39].

The vanadium ion selectivity ($S_{VO^{2+}}$) of the membranes was calculated according to the following formula:

(5) $$S_{{VO}^{2+}}=\frac{\sigma }{P_{{VO}^{2+}}}$$

### 2.5. Preparation of the Chloride-Based VRFB Electrolyte

The working electrolyte used in this study was prepared following the procedure reported in Ref. [9] for the electrolyte indicated as electrolyte B. Briefly, a 2:5 (mol:mol) mixture of aqueous H_2_SO_4_ and HCl was added to oxalic acid dihydrate (C_2_H_2_O_4_·2H_2_O) and vanadium pentoxide (V_2_O_5_), and let to react under stirring for 96 h. After the reaction, the electrolyte was filtered off, and the obtained VO^2+^ solution (OPT_Electrolyte_A) was electrolyzed in a flow cell to reduce half of the V(IV) to V(III). At the end of the electrolysis, the catholyte was mixed with an equal volume of fresh OPT_Electrolyte_A (containing VO^2+^ in H_2_SO_4_/HCl), affording the working electrolyte (OPT_Electrolyte) nominally made up of [V^3+^] = 1.25 M, [VO^2+^] = 1.25 M, [SO_4_^2−^] = 2.50 M, and [Cl^−^] = 6.00 M, with a [V^3+^]/[VO^2+^] ratio equal to 1.0.

### 2.6. Single-Cell VRFB Tests (Charge/Discharge Measurements and EIS)

**Figure 1:**
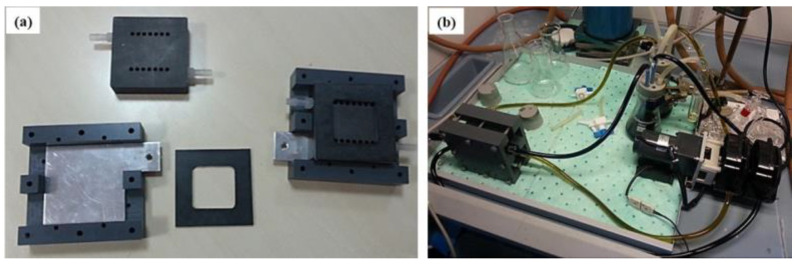
(**a**) Main components and (**b**) set-up of the developed in-house single-cell VRFB.

The charge and discharge tests were carried out using a single-cell VRFB designed in-house [9] using a multichannel battery analyzer (BST8-3, MTI Corporation, Richmond, CA, Canada). The single-cell VRFB, equipped with a cation exchange membrane (Nafion 115 or SPEEK) and a graphite felt electrode (5.0 cm × 5.0 cm, GFD SGL Carbon, Wiesbaden, Germany), was connected to two electrolyte reservoirs (250 mL each) and a dual-head peristaltic pump. In Figure 1a the main components of a single-cell VRFB are shown. Figure 1b displays the set-up of the developed in-house single-cell VRFB.

Before tests, the graphite felt was previously electrochemically oxidized to enhance electrochemical activity and hydrophilicity.

A smart sealing and spacing system, made up of a Viton gasket and a polytetrafluoroethylene (PTFE) spacer, was placed alternately and permitted to vary the compression rate of the carbon electrode, varying the spacer thickness. Two aluminum plates acted as terminal current collectors. The electrolyte circulated through the cell compartment for each half-cell in neoprene tubes using the peristaltic pump under N_2_ atmosphere at a volumetric flow rate in the range of 150–180 mL·min^−1^, corresponding to a linear electrode face velocity of 2.0–2.5 cm·s^−1^. The flow battery was cycled between 1.9 V and 0.7 V, which represent the charge and discharge cut-off voltage, respectively, and at different current densities, starting from 20 mA·cm^−2^ up to 100 mA·cm^−2^, in five steps. The flow rate and the operation temperature were kept constant.

The EIS measurements were carried out by Autolab–PGSTAT302N-FRA32M instrument, (Metrohm AG, Herisau, Switzerland) using a sinusoidal excitation voltage of 5.0 mV by sweeping the frequency from 100 kHz to 0.05 Hz. Impedance spectra were recorded at a state of charge (SoC) of 80% in the absence of the electrolyte flow, and fitted using Metrohm NOVA 2.1 software.

### 2.7. In-House Five-Cell VRFB Stack System

**Figure 2:**
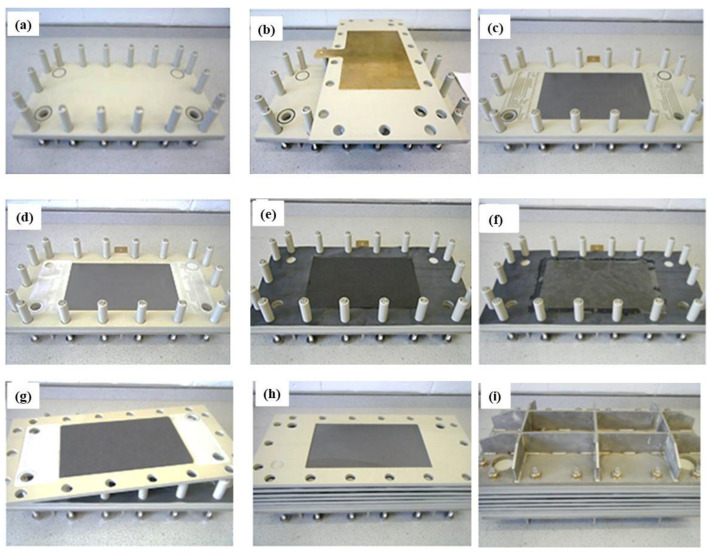
(**a**–**i**) Detailed steps to assemble the in-house five-cell VRFB stack.

The developed in-house five-cell VRFB stack system is shown in Figure S2, in the Supplementary Information. The steps followed to assemble the five-cell stack are shown in Figure 2.

The stack included end cells in polypropylene (Figure 2a), brass current collectors (Figure 2b), PPG86 SGL bipolar plates in polypropylene charged with graphite, cells in polypropylene (Figure 2c,d), and a gasket in fluoropolymer elastomer for the hydraulic sealing of the stack. Uniform circulation of electrolyte species across the active area of the electrode was achieved by using serpentine flow channels. The SPEEK50-0 membrane was sandwiched by two gaskets, and the anode and cathode electrodes were separated hydraulically by carbon felt GFD SGL 4.6 (Figure 2e,f). The stack comprised five cells (Figure 2g,h), and was tightened with a dynamometric torque wrench at 40 Nm using stainless-steel terminal plates and screws (Figure 2i). The hydraulic circuit consisted of PVC pipes, valves, flux meters, and PP tanks.

The ability to precisely set and control the power supply and electronic load ensured proper battery cycling. The power supply (SM6000 series, SM 60-100A,, Delta Elektronika, Zierikzee, The Netherlands) allowed charging the battery in three different modes, as follows: constant current (0–100 A), constant voltage (0–60 V), or constant power (0–6 kW). It was also possible to set the load profile through a specific program. In the same way, the electronic load system (EA-EL9000 EA ELEKTRO-AUTOMATIK) allowed the discharge of the battery at constant current (0–100 A), constant voltage (0–48 V), or constant power (0–4.8 kW).

In Figure S3a,b in the Supplementary Information, the scheme of the in-house five-cell VRFB stack system, and the exploded view of one cell of the VRFB stack system, are reported.

## 3. Results and Discussion

### 3.1. Membranes Characterization

**Figure 3:**
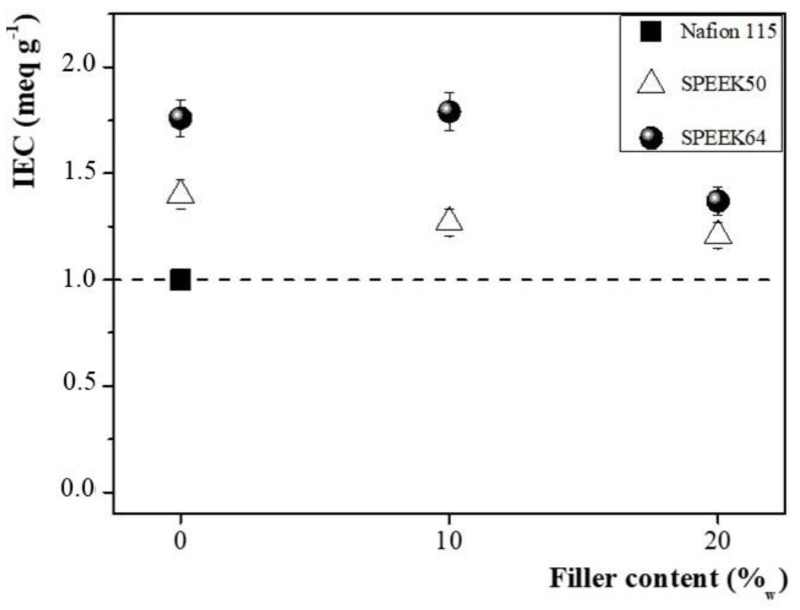
IEC values of SPEEK50 and SPEEK64 membranes without and with different filler content, in comparison to Nafion 115.

IEC indicates the number of exchangeable protons in the membrane, providing, therefore, a direct approximation of the proton conductivity [54]. In Figure 3, the IEC values of unmodified and modified SPEEK membranes are reported and compared to commercial Nafion 115.

In all cases, pristine and SPEEK membranes with filler content revealed IEC values higher than that of commercial Nafion 115. In the prepared SPEEK membranes, the IEC capacity decreased by increasing the filler quantity, probably due to the strong hydrogen bond between the amine groups in the functionalized silica and the sulfonic groups in SPEEK, hindering the conductive pathway [47,51,55,56].

The membrane conductivity depends on the uptake/concentration of ions. Specifically, VRFBs include multi-ionic operating environments, such as H^+^, SO_4_^2−^, Cl^−^, V^3+^/V^2+^, and VO_2_^+^/VO^2+^ ions. For the cation exchange membrane, H^+^ ions are the predominant conducting species. Possible permeation of the other cations, namely V^3+^/V^2+^ and VO_2_^+^/VO^2+^, may cause fast self-discharge and low efficiency of VRFBs [57]. Since vanadium uptake (V_up_) accelerates the degradation of the membrane, the differences in ion conducting groups lead to variations in V_up_ and, thus, in chemical stability [58]. Therefore, for VRFB application, high ionic conductivity plays an important role along with extremely low vanadium ion permeability.

**Figure 4:**
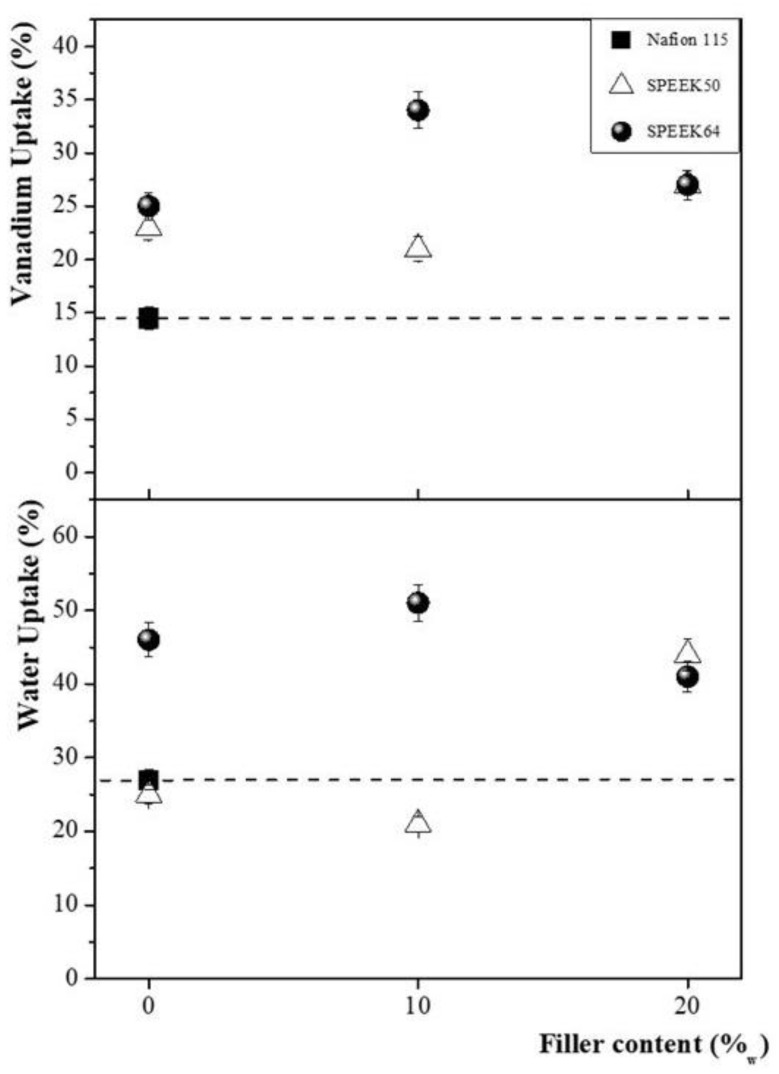
Vanadium uptake and water uptake values of SPEEK50 and SPEEK64 membranes without and with different filler content, in comparison to Nafion 115.

The V_up_ values of prepared SPEEK membranes, compared to those of commercial Nafion 115, are reported in Figure 4.

All prepared SPEEK membranes exhibited a V_up_ capacity higher than Nafion 115. The SPEEK64 membranes containing filler at 10%_w_ and 20%_w_ revealed higher V_up_ values compared to the ones without filler. A different trend was observed for the prepared SPEEK50 membranes. In this case, the highest V_up_ was observed when 20%_w_ of the filler was used. As reported in previous studies [47,49], major interaction between the amino groups of functionalized silica and the sulphonic groups of SPEEK occurs at higher DS; therefore, the different reported behavior is presumably associated with a different interaction between the amino groups of introduced filler and the sulfonic groups of the membranes, depending on the SD and amount of filler. Furthermore, a different arrangement of the polymeric structure depends on both DS and filler content, affecting the values of V_up_ [47,51,59].

In Figure S4 of the Supplementary Information, possible interactions, involved in the vanadium uptake mechanism, between the modified membranes and the vanadyl ions solution are reported.

Figure 4 shows the W_up_ values of the prepared SPEEK membranes, compared to those of commercial Nafion 115. The results reveal that water uptake changes as a function of the DS and the amount of filler introduced, following the same trend reported for V_up_.

Aiming at evaluating the performance of the prepared membranes, the values of permeability, and ionic conductivity before and after the vanadium uptake were determined for each case and compared to Nafion 115 (Table 1). Regarding the permeability, which was in all cases lower than Nafion 115, it presented an increasing trend in dependence on SD. Specifically, SPEEK50-0 gave the lowest value, about two orders of magnitude lower than that of Nafion 115. Once the ionic conductivity (σ) was measured, the values of selectivity to vanadium were determined. As a result, among the prepared membranes, the most selective one resulted in SPEEK50-0 ($S_{VO^{2+}}$ = 49.2 × 10^6^ S·s·cm^−3^), followed by SPEEK64-0 and SPEEK64-20 membranes. Furthermore, the σ_loss_ was determined for all of the cases, finding that SPEEK50-0 gave di the lowest value of σ_loss_ (13%).

Based on the above measurements, SPEEK50-0 and SPEEK64-20 membranes were selected as possible cost-effective alternative membranes to Nafion 115 in single-cell VRFB tests.

### 3.2. Single-Cell VRFB Tests

**Figure 5:**
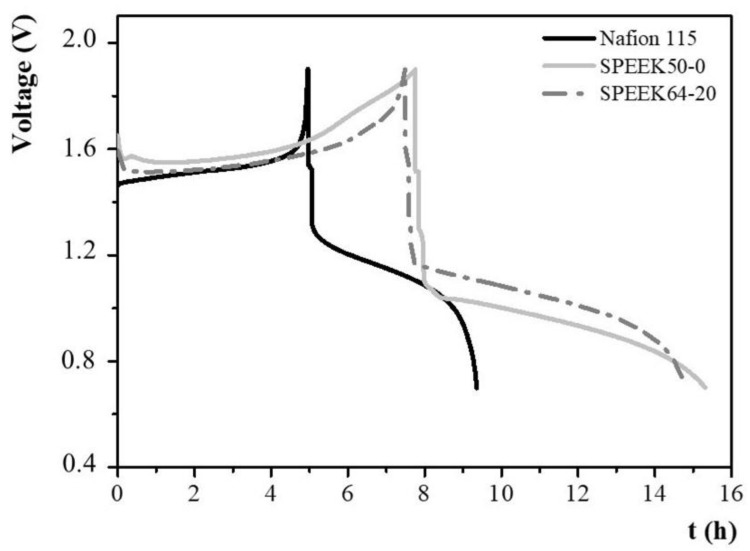
Charge–discharge single-cell VRFB voltage profiles of the prepared SPEEK membranes. Nafion 115 was used as a reference.

The charge–discharge test was performed to determine the cycle performance parameters of single-cell VRFB containing Nafion 115, SPEEK50-0, and SPEEK64-20 membranes. Such tests were carried out at a charge and discharge current density of 20 mA·cm^−2^, using an STD_Electrolyte (Figure 5).

Table 2 lists the results of the charging and discharging tests described above.

Among the studied membranes, as reported in Table 2, SPEEK50-0 showed the longest cycle time (15.3 h), compared to SPEEK64-20 (14.8 h), and Nafion 115 (9.3 h). Such a result in combination with the calculated percentage values of coulombic efficiency in the percentage (CE = Q_discharg_e/Q_charge_ × 100) demonstrated that SPEEK50-0 and SPEEK 64-20, under the cell operation conditions (25 cm^2^ cell area, STD_Electrolyte, charged to 1.9 V by 20 mA cm^−2^, discharged to 0.7 V by 20 mA cm^−2^), were able to accumulate and release a higher charge quantity during the charge and discharge cycle. Specifically, the VRFB single-cell containing SPEEK50-0 and SPEEK64-20 membranes showed higher coulombic efficiencies of 96.1% and 97.1%, respectively, compared to that containing Nafion 115 (85.5%). The single-cell VRFB containing Nafion 115 showed the lowest mean charge voltage and the highest mean discharge voltage. Such results can be attributed to the high ionic conductivity of Nafion 115 as a result of a low specific area resistance. Thus, the thickness of the SPEEK membranes under investigation was opportunely reduced from 70 μm to 45 μm, aiming at reaching conductivity values comparable to those of Nafion 115, considering the conductivity obtained by Equation (3).

Although the single-cell VRFB with SPEEK64-20 membrane showed a higher coulombic efficiency, its production cost still hinders its industrialization. On the other side, the SPEEK50-0 membrane resulted in a good compromise between electrochemical properties and production costs for the set-up of a five-cell VRFB stack system. Further considerations are conducted on a single-cell VRFB with this specific membrane.

### 3.3. Optimization of Single-Cell VRFB

EIS measurements were performed to evaluate the total impedance of the electrochemical system. Furthermore, through the study of the equivalent electrical circuit of the system itself, it was possible to identify the components that more likely affected the single-cell VRFB performance. EIS measurements were carried out in static conditions, namely in the absence of electrolyte flow using GFD SGL 4.6 as carbon felt, and a SoC of 80%. The measurements were performed with Nafion 115 or SPEEK50-0 as membranes, and STD_Electrolyte or OPT_Electrolyte as electrolytes.

**Figure 6:**
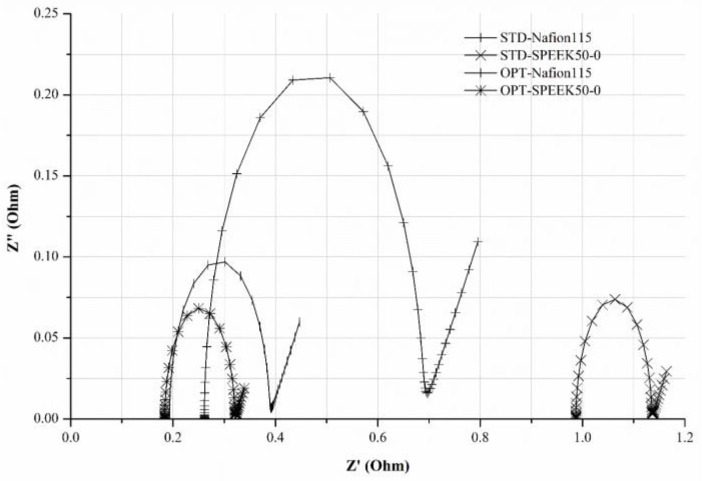
Nyquist plots of EIS using GFD SGL 4.6 as carbon felt, and a SoC of 80%, by varying the membranes and electrolytes to the configuration of the single-cell VRFB.

The Nyquist plots in Figure 6 were obtained, and the most appropriate equivalent electric circuit (Figure S5 in the Supplementary Information) was selected in a way to fit the experimental data. In this specific case, the equivalent electric circuit is composed of the ohmic resistance, R_s_, the charge transfer resistance across the electrode/solution interface, R_ct_, the Warburg diffusion, W, and the constant-phase-element, CPE. The latter represents the electrochemical double layer.

Table 3 summarizes the impedance parameters obtained by fitting the experimental data of the EIS with the opportunely selected equivalent electric circuit.

In the presence of OPT_Electrolyte, a reduced total resistance (R_tot_ = R_s_ + R_ct_) of the VRFB single-cell was observed regardless of the adopted membrane, as reported in Table 3. The single-cell VRFB configuration OPT-SPEEK50-0 showed the lowest value of R_tot_. The values of CPE slightly increased using the OPT_Electrolyte, suggesting that the redox reactions involving V(IV)/V(V) at the electrical double layer (the electrode/electrolyte interface) may be favored.

The highest value of the Warburg diffusion parameter (W) was obtained when the SPEEK50-0 membrane was used in the presence of the OPT_Electrolyte.

Following the previous considerations, the OPT-SPEEK50-0 configuration resulted in it being the most promising one to be adopted in a five-cell VRFB stack system.

To define the appropriate operating configuration of the single-cell VRFB for the construction of the five-cell VRFB stack system, charge–discharge tests were carried out using GFD SGL 4.6 as carbon felt and a compression rate of 0.2, SPEEK50-0 membrane and Nafion115 for comparison, and OPT_Electrolyte. Charge–discharge cycles were performed at different current densities in the range of 20–100 mA cm^−2^ (current in the range of 500–2500 mA with an active area of 25 cm^2^), and the charge and discharge cut-off values equal to 1.9 V and 0.7 V, respectively. The results obtained in charge–discharge tests are reported in Table S1.

**Figure 7:**
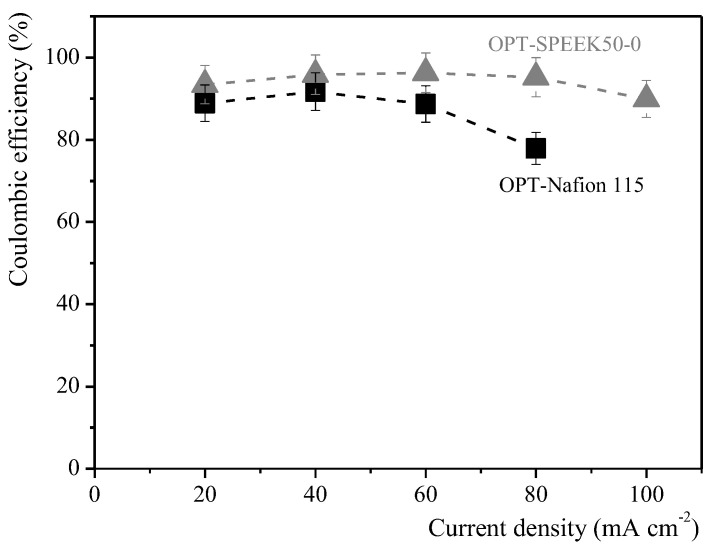
Coulombic efficiency calculated at different current densities by a charge–discharge test, applied to the single-cell VRFB configurations.

As described in Figure 7, the OPT-SPEEK50-0 single-cell configuration showed better electrochemical performances compared to the OPT-Nafion115 one. In the first case, the coulombic efficiency increased constantly from 93.4% up to 96.3%. Further increases in the current density up to 100 mA cm^−2^ caused a decrease in the coulombic efficiency down to 89.9%. However, while ohmic losses were observed in the case of the OPT-Nafion115 configuration with a current density of 100 mA·cm^−2^, this was not the case for the OPT-SPEEK50-0 single-cell configuration. The latter maintained a coulombic efficiency of 89.9%, even when a current density of 100 mA·cm^−2^ was applied.

Based on such considerations, the combination of SPEEK50-0 with the chloride-based electrolyte (OPT) results in a promising configuration for the construction of a five-cell VRFB stack system.

### 3.4. Five-Cell VRFB Stack System

A five-cell VRFB stack system was assembled starting from the OPT-SPEEK50-0 configuration as optimized in the previous paragraph. Table 4 lists the details of operating parameters for the charge/discharge tests on the in-house five-cell VRFB stack system.

The five-cell VRFB stack system was, firstly, conditioned by a charging step at 20 mA·cm^−2^ for 20 min before the discharge step. Such a value of current density was selected as a way to simulate the operating condition of the single-cell VRFB. During this conditioning step, the carbon felts were activated through electrochemical oxidation, which allowed them to reach a lower activation overpotential in the following steps, thanks to the formation of hydrophilic functional groups on the carbon felt surface.

To verify the battery performance at different operating conditions, the first charge–discharge cycle was carried out at a current density of 20 mA·cm^−2^. Afterward, two charge–discharge cycles were performed at 50 mA·cm^−2^, followed by one at 80 mA·cm^−2^, and the last at 100 mA·cm^−2^.

**Figure 8:**
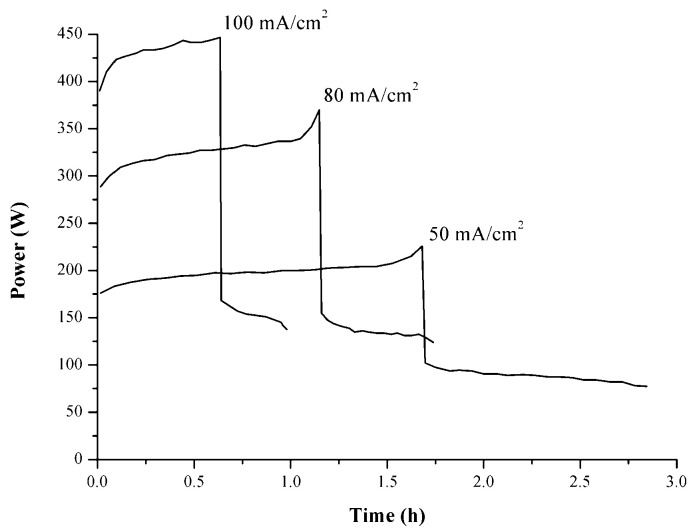
Charge–discharge power profiles (vs. time) of the in-house produced five-cell VRFB stack system at different current densities.

The charge–discharge power profiles for the dependence on time at different current densities, i.e., 50, 80, and 100 mA·cm^−2^, are reported in Figure 8.

The in-house produced five-cell VRFB stack system based on SPEEK50-0 membrane and chloride-based electrolyte displayed encouraging preliminary electrochemical results. The membrane maintained the functionality of the system even at current density values of 100 mA·cm^−2^. Nevertheless, the best results were obtained when a current density of 20 mA·cm^−2^ was applied (CE = 94.2%), since a slight loss in the CE was observed with increasing the values of current density (Table 5).

## 4. Conclusions

In conclusion, the physicochemical features of different SPEEK membranes were optimized in a single-cell vanadium flow battery (VRFB), and, subsequently, employed in a large-scale five-cell VRFB stack system.

In the first part of the present work, the electrochemical performance of a single-cell VRFB was tested for dependence on the type of ion exchange membrane (SPEEK membranes vs. Nafion 115), and the composition of the adopted electrolyte.

Particular attention was paid to optimizing the SD and evaluating the effect of silica gel functionalized with 3-aminopropyl added as a filler. It was found that among the prepared SPEEK membranes, SPEEK50-0 and SPEEK64-20 presented good durability, permeability, and selectivity to vanadium.

Such membranes were successfully integrated into a single-cell VRFB using sulfuric acid as an electrolyte. Based on charge–discharge cycles, improved performance in terms of coulombic efficiency at 20 mA·cm^−2^, compared to Nafion 115, was achieved (96.1% for SPEEK50-0; 97.1% for SPEEK64-20; 85.5% for Nafion 115).

A decrease in the total resistance of the system was observed through EIS measurements, regardless of the type of membrane adopted when a chloride-based electrolyte was used. This result, combined with the intrinsic higher energy density of this electrolyte, makes its use even more advantageous. A five-cell VRFB stack system was assembled, using the optimized chloride-based electrolyte and selecting SPEEK50-0 as a membrane over SPEEK64-20, considering the higher availability of the former. The resulting energy stack system showed a value of coulombic efficiency (94.5%) similar to that obtained for the single-cell VRFB (96.1%), at 20 mA·cm^−2^. Interestingly, the membrane remained undamaged, even when a current density of 100 mA·cm^−2^ was applied.

The results of this study pave the way for the further development of this class of membranes, aiming at extending the plethora of membranes to be selectively applied in VRFB systems for long-term energy storage.

## Figures and Tables

**Table 1:** Data of permeability, selectivity, initial- and post-vanadium uptake conductivity, with the corresponding percentage of conductivity loss, related to the prepared membranes, in comparison to values of Nafion 115.

Membrane	$P_{{VO}^{2+}}$ (cm^2^·s^−1^)	$S_{VO^{2+}}$ (10^6^ S·s·cm^−3^)	σ (S·cm^−1^)	σ_afterVuptake_ (S·cm^−1^)	σ_loss_ (%)
Nafion 115	1.52 10^−8^	5.9	9.00·10^−2^	5.52·10^−2^	38
SPEEK50-0	3.23 10^−10^	49.2	1.59·10^−2^	1.39·10^−2^	13
SPEEK50-10	3.55 10^−10^	17.2	1.59·10^−2^	6.12·10^−3^	62
SPEEK50-20	1.22 10^−9^	15.9	2.60·10^−2^	1.94·10^−2^	25
SPEEK64-0	8.60 10^−10^	25.3	2.18·10^−2^	9.25·10^−3^	58
SPEEK64-10	2.60 10^−9^	18.8	4.90·10^−2^	3.46·10^−2^	29
SPEEK64-20	9.09 10^−10^	27.5	2.00·10^−2^	1.60·10^−2^	20

**Table 2:** Charge–discharge performance parameters * of single-cell VRFB using SPEEK50-0 and SPEEK64-20 membranes, compared to Nafion 115.

Membrane	Cycle Time (h)	Mean Charge Voltage (V)	Mean Discharge Voltage (V)	Coulombic Efficiency (%)
Nafion 115	9.35	1.53	1.12	85.5
SPEEK50-0	15.32	1.64	0.93	96.1
SPEEK64-20	14.79	1.58	1.02	97.1

* Cell operation conditions: 25 cm^2^ cell area, STD_Electrolyte, charged to 1.9 V by 20 mA cm^−2^, discharged to 0.7 V by 20 mA cm^−2^.

**Table 3:** Impedance parameters obtained from Nyquist plots of single-cell VRFBs, using different membranes (Nafion 115 and SPEEK50-0) and electrolytes (STD and OPT).

Configuration	R_s_ (mΩ)	R_ct_ (mΩ)	CPE (mF)	W (S s^0.5^)	R_tot_ (mΩ)
STD-Nafion115	261	425	8.5	36.5	686
STD-SPEEK50-0	987	147	7.0	136	1134
OPT-Nafion115	193	195	9.8	66.7	388
OPT-SPEEK50-0	184	136	11.5	210	320

**Table 4:** Details of operating parameters for the charge/discharge tests on the five-cell VRFB stack.

Operating Parameter	Details
Electrolyte temperature	25 ± 2 °C
Optimized composition of the electrolyte	[V^3+^] = 1.25 M, [VO^2+^] = 1.25 M [SO_4_^2−^] = 2.50 M [Cl^−^] = 6.00 M
Anode face velocity	1.5 cm·s^−1^
Cathode face velocity	1.5 cm·s^−1^
Charge method	Constant current
Charge current density (cycle 1)	20 mA·cm^−2^
Charge current density (cycle > 1)	20–60 mA·cm^−2^
Top-of-charge cut-off voltage (per cell)	1.7–2.0 V
Discharge method	Constant current
Discharge current density (cycle 1)	20–100 mA·cm^−2^
Discharge current density (cycle > 1)	20–100 mA·cm^−2^
Bottom-of-discharge cut-off voltage (per cell)	0.7 V
Carbon felt	GFD SGL 4.6
Compression rate	0.2
Electrode area	459 cm^2^ (17 cm × 27 cm)
Bipolar Plate	PPG86 SGL
Membrane	Optimized SPEEK

**Table 5:** Electrochemical performance parameters obtained after each charge–discharge cycle performed on the five-cell VRFB stack in the OPT-SPEEK50-0.

Cycle	Current Density (mA/cm^2^)	Current (A)	Electrolyte Volume (L)	Discharge Capacity (Ah/L)	Coulombic Efficiency (%)	Mean Charge Voltage (V)	Mean Discharge Voltage (V)
1 *	20	9.2	3	-	-	8.7	3.9
2	20	9.2	3	6.7	94.5	8.6	3.9
3	50	23.0	3	7.1	87.1	8.8	3.8
4	50	23.0	5	6.2	87.3	8.8	3.8
5	80	36.7	5	5.5	79.5	8.9	3.6
6	100	44.3	5	4.9	74.0	9.6	3.4

* Conditioning step.

## Data Availability

The original contributions presented in the study are included in the article/Supplementary Material, further inquiries can be directed to the corresponding authors.

## Supplementary Materials

The following supporting information can be downloaded at: https://www.mdpi.com/article/10.3390/membranes14080176/s1, Figure S1. Chemical structures of (a) Nafion, (b) PEEK, (c) SPEEK, and (d) composited SPEEK; Figure S2. The in-house five-cell VRFB stack; Figure S3: (a) Scheme of the five-cell VRFB stack; (b) exploded view of a VRFB single-cell; Figure S4: Possible interactions involved in the vanadium uptake process; dotted lines represent hydrogen bonding; Figure S5: The proposed model of the equivalent electric circuit for the VRFB single-cell; Table S1: Charge–discharge performance parameters of evaluated single-cell VRFB configurations.
