# Supplementary material for: Optimized Sulfonated Poly(Ether Ether Ketone) Membranes for In-House Produced Small-Sized Vanadium Redox Flow Battery Set-Up
Source: Membranes (Basel). 2024 Aug 14;14(8):176. doi: 10.3390/membranes14080176 (PMC11356608; doi:10.3390/membranes14080176)
Supplement: Supplementary file 1 [file membranes-14-00176-s001.zip › membranes-3138242-supplementary.pdf]

membranes

Supplementary Information

Optimized Sulfonated Poly(Ether Ether Ketone) Membranes for In-House Produced Small-Sized Vanadium Redox-Flow Battery Set-up

Antonino Rizzuti [1,*], Elena Dilonardo [1,2,*], Gennaro Cozzolino [3], Fabio Matera [4], Alessandra Carbone [5], Biagia Musio [1], and Piero Mastrorilli [1,*]

1 Department of Civil, Environmental, Land, Building and Chemical Engineering (DICATECh), Politecnico di Bari, Via E. Orabona 4, 70125, Bari (BA), Italy.
2 Institute of Nanotechnology, CNR-NANOTEC, Via G. Amendola, 122, 70125, Bari (BA), Italy.
3 InResLab, Via Baione, 70043, Monopoli (BA), Italy.
4 Institute of Microelectronics and Microsystems, CNR- IMM, Catania HQ, VIII strada n. 5, 95100, Zona Industriale – Catania (CT), Italy.
5 Institute for Advanced Energy Technologies, CNR-ITAE, Salita S. Lucia Sopra Contesse 5, 98126, Messina (ME), Italy.
* Correspondence: antonino.rizzuti@poliba.it (A. R.); elena.dilonardo@cnr.it (E. D.); pietro.mastrorilli@poliba.it (P. M.)

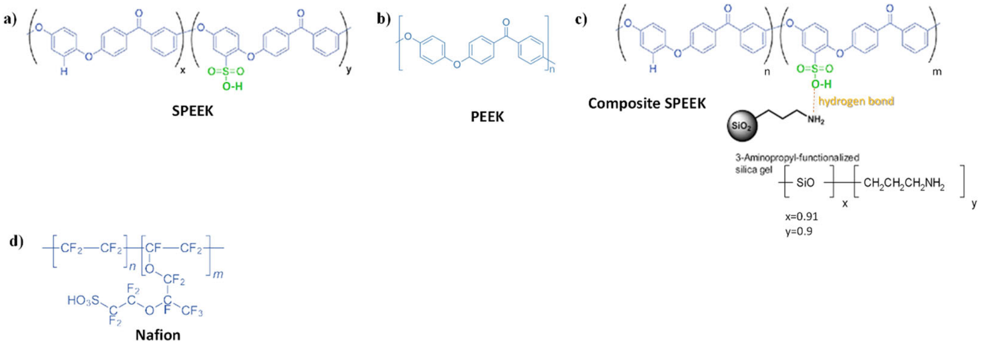

Figure S1. Chemical structures of **(a)** Nafion, **(b)** PEEK, **(c)** SPEEK, and (**d)** composited SPEEK.

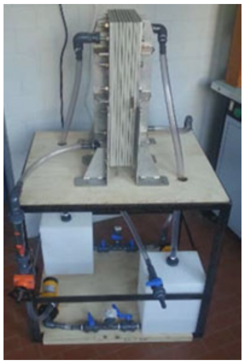

Figure S2. The in-house VRFB five-cell stack.

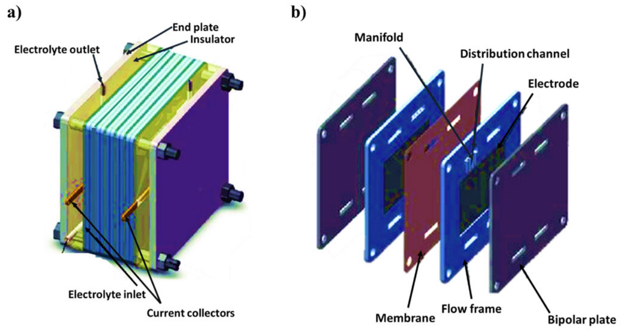

Figure S3. **(a)** Scheme of the five-cell VRFB stack; **(b)** exploded view of a single cell VRFB.

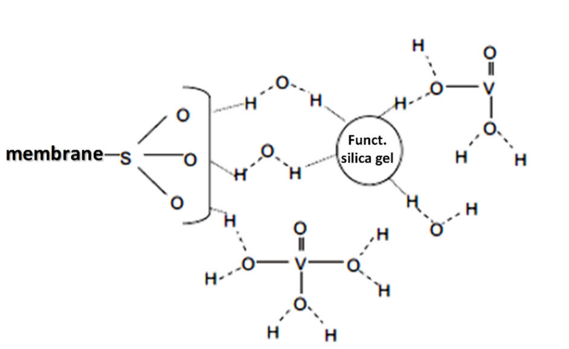

Figure S4. Possible interactions involved in the vanadium uptake process; dotted lines represent hydrogen bonding.

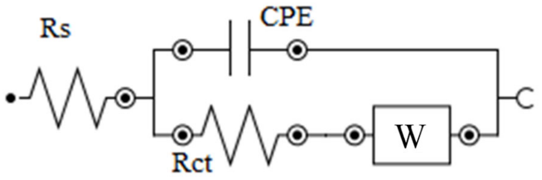

Figure S5. The proposed model of the equivalent electric circuit for the single cell VRFB.

Table S1. Charge-discharge performances parameters of evaluated single-cell VRFB configurations.

Configurations	Current Density (mA cm^{-2})	Charge Cycle Time (h)	Discharge Cycle Time (h)	Mean Charge Voltage (V)	Mean Discharge Voltage (V)	Coulombic Efficiency (%)
OPT-Nafion115	20	9.48	8.42	1.52	1.21	88.9
	40	4.47	4.09	1.51	1.09	91.7
	60	2.67	2.37	1.72	0.98	88.7
	80	1.41	1.10	1.83	0.86	77.9
	100	-	-	-	-	-
OPT-SPEEK50_0	20	8.96	8.38	1.44	1.19	93.4
	40	4.20	4.02	1.65	1.07	95.8
	60	2.66	2.56	1.72	0.97	96.3
	80	1.86	1.76	1.79	0.89	95.2
	100	1.22	1.10	1.83	0.82	89.9

Disclaimer/Publisher's Note: The statements, opinions and data contained in all publications are solely those of the individual author(s) and contributor(s) and not of MDPI and/or the editor(s). MDPI and/or the editor(s) disclaim responsibility for any injury to people or property resulting from any ideas, methods, instructions or products referred to in the content.
